# Improving the accessibility and transferability of machine learning algorithms for identification of animals in camera trap images: MLWIC2

**DOI:** 10.1002/ece3.6692

**Published:** 2020-09-16

**Authors:** Michael A. Tabak, Mohammad S. Norouzzadeh, David W. Wolfson, Erica J. Newton, Raoul K. Boughton, Jacob S. Ivan, Eric A. Odell, Eric S. Newkirk, Reesa Y. Conrey, Jennifer Stenglein, Fabiola Iannarilli, John Erb, Ryan K. Brook, Amy J. Davis, Jesse Lewis, Daniel P. Walsh, James C. Beasley, Kurt C. VerCauteren, Jeff Clune, Ryan S. Miller

**Affiliations:** ^1^ Quantitative Science Consulting, LLC Laramie WY USA; ^2^ Department of Zoology and Physiology University of Wyoming Laramie WY USA; ^3^ Computer Science Department University of Wyoming Laramie WY USA; ^4^ Minnesota Cooperative Fish and Wildlife Research Unit Department of Fisheries, Wildlife and Conservation Biology University of Minnesota St. Paul MN USA; ^5^ Wildlife Research and Monitoring Section Ontario Ministry of Natural Resources and Forestry Peterborough ON Canada; ^6^ Range Cattle Research and Education Center, Wildlife Ecology and Conservation University of Florida Ona FL USA; ^7^ Colorado Parks and Wildlife Fort Collins CO USA; ^8^ Wisconsin Department of Natural Resources Madison WI USA; ^9^ Conservation Sciences Graduate Program University of Minnesota St. Paul MN USA; ^10^ Forest Wildlife Populations and Research Group Minnesota Department of Natural Resources Grand Rapids MN USA; ^11^ Department of Animal and Poultry Science University of Saskatchewan Saskatoon SK Canada; ^12^ National Wildlife Research Center United States Department of Agriculture Fort Collins CO USA; ^13^ College of Integrative Sciences and Arts Arizona State University Mesa AZ USA; ^14^ US Geological Survey National Wildlife Health Center Madison WI USA; ^15^ Savannah River Ecology Laboratory Warnell School of Forestry and Natural Resources University of Georgia Aiken SC USA; ^16^ National Wildlife Research Center United States Department of Agriculture, Animal and Plant Health Inspection Service Fort Collins CO USA; ^17^ OpenAI San Francisco CA USA; ^18^ Center for Epidemiology and Animal Health United States Department of Agriculture Fort Collins CO USA

**Keywords:** computer vision, deep convolutional neural networks, image classification, machine learning, motion‐activated camera, R package, remote sensing, species identification

## Abstract

Motion‐activated wildlife cameras (or “camera traps”) are frequently used to remotely and noninvasively observe animals. The vast number of images collected from camera trap projects has prompted some biologists to employ machine learning algorithms to automatically recognize species in these images, or at least filter‐out images that do not contain animals. These approaches are often limited by model transferability, as a model trained to recognize species from one location might not work as well for the same species in different locations. Furthermore, these methods often require advanced computational skills, making them inaccessible to many biologists. We used 3 million camera trap images from 18 studies in 10 states across the United States of America to train two deep neural networks, one that recognizes 58 species, the “species model,” and one that determines if an image is empty or if it contains an animal, the “empty‐animal model.” Our species model and empty‐animal model had accuracies of 96.8% and 97.3%, respectively. Furthermore, the models performed well on some out‐of‐sample datasets, as the species model had 91% accuracy on species from Canada (accuracy range 36%–91% across all out‐of‐sample datasets) and the empty‐animal model achieved an accuracy of 91%–94% on out‐of‐sample datasets from different continents. Our software addresses some of the limitations of using machine learning to classify images from camera traps. By including many species from several locations, our species model is potentially applicable to many camera trap studies in North America. We also found that our empty‐animal model can facilitate removal of images without animals globally. We provide the trained models in an R package (MLWIC2: Machine Learning for Wildlife Image Classification in R), which contains Shiny Applications that allow scientists with minimal programming experience to use trained models and train new models in six neural network architectures with varying depths.

## Graphical abstract

We provide an R package (MLWIC2), which includes two models with 97% validation accuracy, one that recognizes 58 animal species from camera trap images and another that removes empty images. The R package includes Shiny Applications, allowing users to apply trained models and train new models using a point‐and‐click interface.

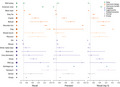

## 1 INTRODUCTION

Motion‐activated wildlife cameras (or “camera traps”) are frequently used to remotely observe wild animals, but images from camera traps must be classified to extract their biological data (O’Connell, Nichols, & Karanth, 2011). Manually classifying camera trap images is an encumbrance that has prompted scientists to use machine learning to automatically classify images (Norouzzadeh et al., 2018; Willi et al., 2019), but this approach has limitations.

We address two major limitations of using machine learning to automatically classify animals in camera trap images. First, machine learning models trained to recognize species from one location and in one camera trap setup might perform poorly when applied to images from camera traps in different conditions (i.e., these models can have low “out‐of‐sample” accuracy; Schneider, Greenberg, Taylor, & Kremer, 2020). This transferability, or generalizability, problem is thought to arise because different locations have different backgrounds (the part of the picture that is not the animal) and most models evaluate the entire image, including the background (Beery, Morris, & Yang, 2019; Miao et al., 2019; Norouzzadeh et al., 2019; Terry, Roy, & August, 2020; Wei, Luo, Ran, & Li, 2020). By including images from 18 different studies in North America, our objective was to train models with more variation in the backgrounds associated with each species. Furthermore, by training an additional model that distinguishes between images with and without animals, we provide an option that could be broadly applicable to camera trap studies worldwide.

Second, the use of machine learning in camera trap analysis is often limited to computer scientists, yet the need for image processing exceeds the availability of computer scientists in wildlife research. For example, several researchers have provided excellent Python repositories for using computer vision to analyze camera trap images (Beery et al., 2019; Beery, Wu, Rathod, Votel, & Huang, 2020; Norouzzadeh et al., 2018; Schneider et al., 2020). These software packages enable programmers to use and train models to detect, classify, and evaluate the behavior of animals in camera trap images. However, these packages require extensive programming experience in Python, a skill which is often lacking from wildlife research teams. To facilitate the use of this type of model by biologists with minimal programming experience, Machine Learning for Wildlife Image Classification (MLWIC2) includes an option to train and use models in user‐friendly Shiny Applications (Chang, Cheng, Alaire, Xie, & McPherson, 2019), allowing users to point‐and‐click instead of using a command line. This facilitates easier site‐specific model training when our models do not perform to expectations.

## 2 MATERIALS AND METHODS

### 2.1 Camera trap images

Images were collected from 18 studies using camera traps in 10 states in the United States of America (California, Colorado, Florida, Idaho, Minnesota, Montana, South Carolina, Texas, Washington, and Wisconsin; Appendix S1). Images were either classified by a single wildlife expert or classified independently by two biologists, with discrepancies settled by a third. An image was classified as containing an animal if it contained any part of an animal. Our initial dataset included 6.3 million images but was unbalanced with most images from a few species (e.g., 51% of all images were *Bos taurus*). We rebalanced the number of images by species and site to ensure that no one species or site dominated the training process. Previous work suggested that training a model with 100,000 images per species produces good performance (Tabak et al., 2019); therefore, we limited the number of images for a single species from one location to 100,000. When >100,000 images for a single species existed at one location, we randomly selected 100,000 of these images to include in the training/testing dataset. After rebalancing the data, we had a total of 2.98 million images; 90% were randomly selected for training, while 10% were used for testing. Images used in this study were either already a part of or were added to the North American Camera Trap Images dataset (lila.science/datasets/nacti; Tabak et al., 2019). Images from Canada were not used for training but were used to evaluate model transferability as an out‐of‐sample dataset.

### 2.2 Training models

We trained deep convolutional neural networks using the ResNet‐18 architecture (He, Zhang, Ren, & Sun, 2016) in the TensorFlow framework (Adabi et al., 2016) on a high‐performance computing cluster, “Teton” (Advanced Research Computing Center, 2018). Models were trained for 55 epochs, with a ReLU activation function at every hidden layer and a softmax function in the output layer, mini‐batch stochastic gradient descent with a momentum hyperparameter of 0.9 (Goodfellow, Bengio, & Courville, 2016), a batch size of 256 images, and learning rates and weight decays that varied by epoch number (described in Appendix S2). We trained a species model, which contained classes for 58 species or groups of species and one class for empty images (Table 1). We also trained an empty‐animal model that contained only two classes, one for images containing an animal, and the other for images without animals.

**TABLE 1:** Comparison of validation accuracy (accuracy on the withheld dataset) using different architectures

Architecture	Validation accuracy
ResNet‐18	96.8
DenseNet‐121	95.9
VGG‐22	88.6
GoogleNet‐32	88.1
AlexNet‐8	85.4
NiN‐16	84.3

### 2.3 Model validation and transferability

We first evaluated our trained models by applying them to predicting species in the 10% of images that were withheld from training. Models were evaluated for each species using the recall, top‐5 recall, and precision, which are values summarizing the number of true positives (TPs), false positives (FPs), and false negatives (FNs):

$$\text{Recall}=\frac{\text{TP}}{\text{TP}+\text{FN}}$$

$$\text{Precision}=\frac{\text{TP}}{\text{TP}+\text{FP}}.$$

As recall is the proportion of images of each species that were correctly classified, top‐5 recall is the proportion of images for each species in which one of the model's top five guesses is the correct species. We also calculated confidence intervals for recall and precision rates (Appendix S3). To evaluate transferability of the model, we conducted out‐of‐sample validation by applying our trained models to images from locations where the model was not trained. We evaluated the species model using four out‐of‐sample datasets from North America: the Caltech Camera Traps dataset (Beery, Van Horn, & Perona, 2018), the ENA24‐detection dataset (Yousif, Kays, & He, 2019), the Saskatchewan, Canada dataset from this study, and the Missouri Camera Traps dataset (Zhang, He, Cao, & Cao, 2016). The empty‐animal model was tested using the Wellington Camera Traps dataset from New Zealand (Anton, Hartley, Geldenhuis, & Wittmer, 2018), the Snapshot Serengeti dataset from Tanzania (Swanson et al., 2015), and the Snapshot Karoo dataset from South Africa (http://lila.science/datasets/snapshot‐karoo).

To evaluate the effect of using multiple training datasets on model generalizability, we iteratively trained models using varying numbers of datasets (i.e., 1 dataset, 3 datasets, 6 datasets, … all 18 datasets) and tested the model on the out‐of‐sample datasets.

### 2.4 R package development

MLWIC2 was developed using the R packages Shiny (Chang et al., 2019) and ShinyFiles (Pedersen, Nijs, Schaffner, & Nantz, 2019) so the user can choose to either use a programming console or a graphical user interface. Users can navigate to locations on their computer using a browser window instead of specifying paths. The package can classify images at a rate of 2,000 images per minute on a laptop with 16 gigabytes of random‐access memory and without a graphics processing unit. MLWIC2 will optionally write the top guess from each model and confidence associated with these guesses to the metadata of the original image file. The function “write_metadata” and the associated R Shiny Application uses Exiftool (Harvey, 2016) to accomplish this. In addition, if scientists have labeled images, MLWIC2 has a Shiny app that allows users to train a new model to recognize species using one of six different convolutional neural network architectures (AlexNet, DenseNet, GoogLeNet, NiN, ResNet, and VGG) with different numbers of layers. We also trained models in these other architectures for comparison. Note that the time required to train a model depends on the number of images used for training and computing resources; operating MLWIC2 on a high‐performance computing cluster requires programming experience.

## 3 RESULTS

**FIGURE 1:**
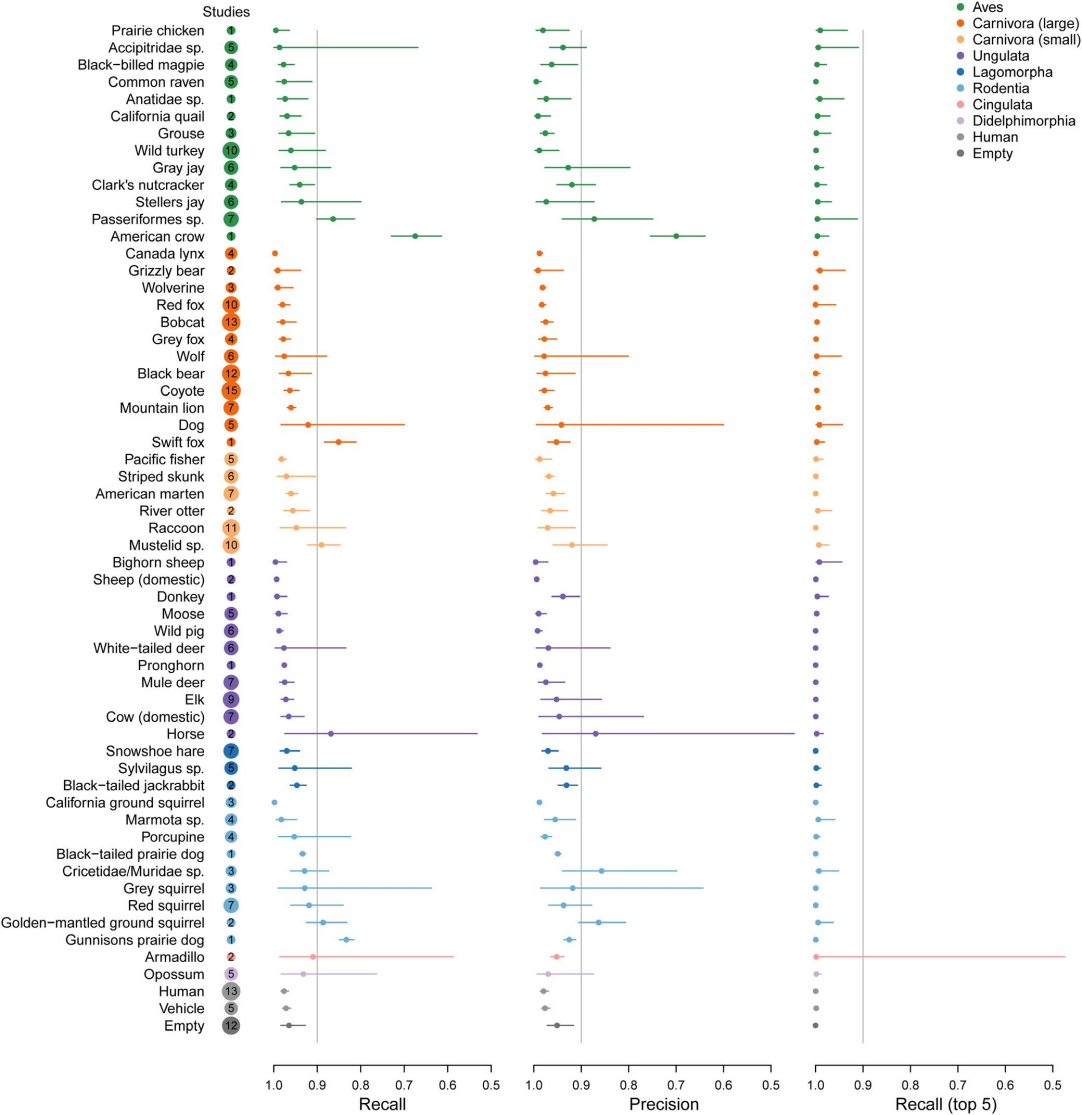
Within sample validation of the species model revealed high recall and precision for most species. Median values across datasets are presented along with 95% confidence intervals. The number of datasets for each species is included in the circle next to the species name (circle sizes are proportional to the number of datasets containing each species)

**FIGURE 2:**
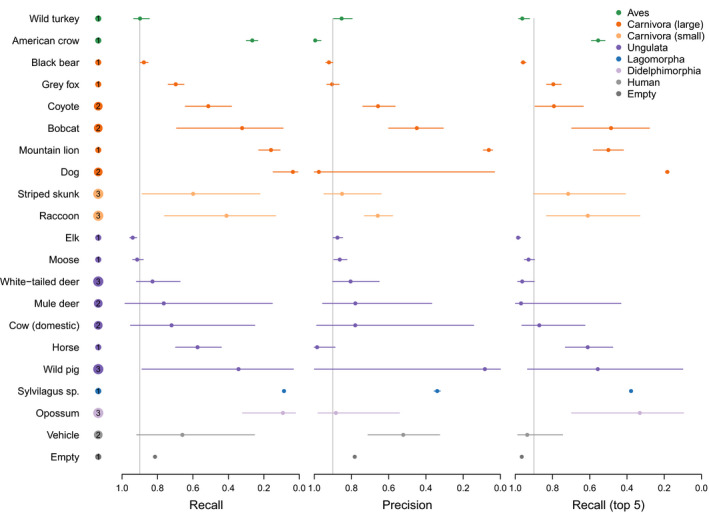
Species model out‐of‐sample validation revealed variable recall and precision rates across species. Median values across datasets are presented along with 95% confidence intervals. The number of datasets for each species is included in the circle next to the species name

**FIGURE 3:**
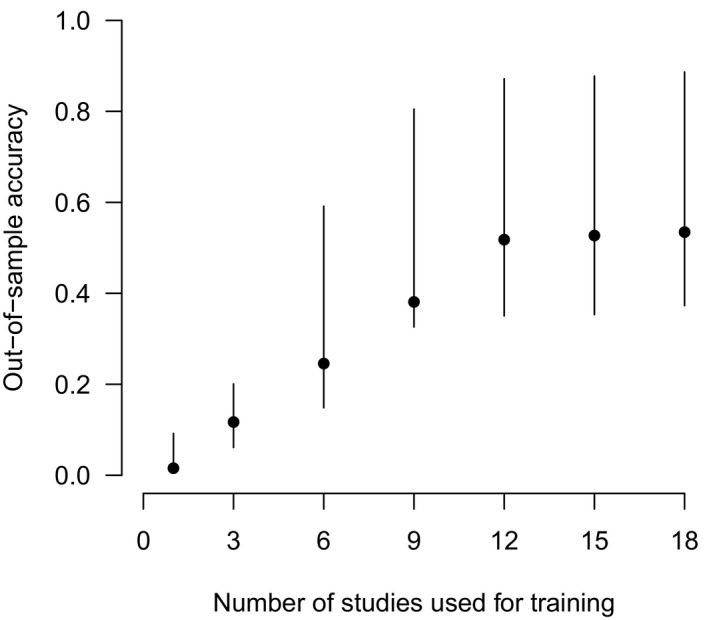
Models became more generalizable (i.e., out‐of‐sample accuracy increased) as the number of datasets used to train the model increased. Points represent median accuracy across out‐of‐sample datasets and lines connect the minimum and maximum of the 95% quantiles for accuracy values across these datasets

We found the highest validation accuracy (within sample validation) using ResNet‐18 (Table 1), for which we found an overall accuracy of 96.8% for the species model and 97.3% for the empty‐animal model. Several species (6 of 11) had recall of >95% with fewer than 2,000 images used for training (Table 2; Figure 1). A confusion matrix (Appendix S4) depicts how all images of each species were classified by the species model. When evaluated on out‐of‐sample images, the species model accuracy ranged from 36.3% to 91.3% (Table 3), with top‐5 accuracy ranging from 65.2% to 93.8% (Figure 2), and the empty‐animal model accuracy ranged from 90.6% to 94.1% (Table 3). When we iteratively trained the model on varying numbers of datasets, we found that accuracy on out‐of‐sample images increased with the number of datasets used to train the model (Figure 3).

**TABLE 2:** Mean recall and precision rates (along with 95% confidence intervals) for predicting species using the species model on the validation dataset (the 10% of images that were withheld from training)

Class name (scientific name)	Number of training images	Recall	Precision
Accipitridae family (Accipitridae)	1,511	0.91 (0.67, 1)	0.94 (0.89, 0.97)
American crow (*Corvus brachyrhynchos*)	2,522	0.67 (0.61, 0.73)	0.7 (0.64, 0.75)
American marten (*Martes americana*)	51,081	0.96 (0.95, 0.97)	0.96 (0.94, 0.97)
Anatidae family (Anatidae)	1,071	0.97 (0.92, 0.99)	0.97 (0.92, 0.99)
Armadillo (Cingulata)	8,947	0.94 (0.59, 0.99)	0.95 (0.94, 0.96)
Bighorn sheep (*Ovis canadensis*)	1,189	1 (0.97, 1)	1 (0.97, 1)
Black bear (*Ursus americanus*)	111,426	0.97 (0.91, 0.99)	0.99 (0.91, 0.99)
Black‐billed magpie (*Pica hudsonia*)	2,770	0.98 (0.95, 0.99)	0.96 (0.91, 0.99)
Black‐tailed jackrabbit (*Lepus californicus*)	5,617	0.95 (0.93, 0.96)	0.93 (0.91, 0.95)
Black‐tailed prairie dog (*Cynomys ludovicianu*s)	43,999	0.93 (0.93, 0.94)	0.95 (0.94, 0.96)
Bobcat (*Lynx rufus*)	31,634	0.96 (0.95, 0.99)	0.97 (0.96, 0.98)
California ground squirrel (*Otospermophilus beecheyi*)	30,301	1 (1, 1)	0.99 (0.98, 0.99)
California quail (*Callipepla californica*)	2,046	0.97 (0.94, 0.99)	0.99 (0.97, 1)
Canada lynx (*Lynx canadensis*)	15,119	1 (0.99, 1)	0.99 (0.98, 0.99)
Cattle (*Bos taurus*)	269,963	0.97 (0.93, 0.98)	0.98 (0.77, 0.99)
Clark's nutcracker (*Nucifraga columbiana*)	2,785	0.94 (0.91, 0.96)	0.92 (0.87, 0.95)
Common raven (*Corvus corax*)	21,134	0.99 (0.91, 0.99)	0.99 (0.98, 1)
Coyote (*Canis latrans*)	41,512	0.96 (0.94, 0.98)	0.97 (0.96, 0.99)
Cricetidae and Muridae families	1,254	0.93 (0.87, 0.96)	0.83 (0.7, 0.94)
Dog (*Canis familiaris*)	1,136	0.82 (0.7, 0.98)	0.78 (0.6, 0.99)
Domestic sheep (*Ovis aries*)	16,340	0.99 (0.99, 1)	0.99 (0.99, 1)
Donkey (*Equus asinus*)	2,403	0.99 (0.97, 1)	0.94 (0.9, 0.96)
Elk (*Cervus canadensis*)	112,389	0.97 (0.95, 0.98)	0.99 (0.86, 0.99)
Empty (no animal)	907,096	0.97 (0.93, 0.98)	0.95 (0.92, 0.97)
Fisher (*Pekania pennanti*)	7,697	0.98 (0.97, 0.99)	0.99 (0.96, 1)
Golden‐mantled ground squirrel (*Callospermophilus lateralis*)	1,587	0.89 (0.83, 0.92)	0.86 (0.81, 0.91)
Grey fox (*Urocyon cinereoargenteus*)	16,094	0.98 (0.96, 0.99)	0.97 (0.95, 0.99)
Grey jay (*Perisoreus canadensis*)	3,776	0.97 (0.87, 0.98)	0.94 (0.8, 0.98)
Grey squirrel (*Sciurus carolinensis*)	24,677	0.98 (0.64, 0.99)	0.98 (0.64, 0.99)
Grizzly bear (*Ursus arctos horribilis*)	843	0.99 (0.94, 1)	0.99 (0.94, 1)
Gunnison's prairie dog (*Cynomys gunnisoni*)	17,393	0.83 (0.82, 0.85)	0.93 (0.91, 0.94)
Horse (*Equus ferus*)	3,644	0.94 (0.53, 0.97)	0.95 (0.45, 0.98)
Human (*Homo sapiens*)	139,983	0.98 (0.97, 0.98)	0.98 (0.97, 0.99)
Marmota genus (*Marmota* spp.)	1,497	0.98 (0.95, 0.99)	0.95 (0.91, 0.98)
Moose (*Alces alces*)	11,741	0.99 (0.97, 1)	0.99 (0.97, 1)
Mountain lion (*Puma concolor*)	13,900	0.96 (0.95, 0.97)	0.97 (0.96, 0.98)
Mule deer (*Odocoileus hemionus*)	91,068	0.98 (0.95, 0.99)	0.98 (0.93, 0.99)
Opossum (Didelphimorphia)	5,782	0.94 (0.76, 0.98)	0.97 (0.87, 0.99)
Other grouse (Tetraoninae)	4,237	0.97 (0.91, 0.99)	0.98 (0.96, 0.99)
Other mustelids (Mustelidae)	2,467	0.89 (0.85, 0.92)	0.91 (0.85, 0.96)
Other passerine birds (Passeriformes)	3,363	0.86 (0.81, 0.9)	0.88 (0.75, 0.94)
Porcupine (Erethizontidae and Hystricidae)	6,608	0.97 (0.82, 0.99)	0.98 (0.96, 0.98)
Prairie chicken (*Tympanuchus cupido*)	815	1 (0.96, 1)	0.98 (0.93, 1)
Pronghorn (*Antilocapra americana*)	57,953	0.98 (0.97, 0.98)	0.99 (0.98, 0.99)
Raccoon (*Procyon lotor*)	51,439	0.9 (0.83, 0.99)	0.93 (0.91, 0.99)
Red fox (*Vulpes vulpes*)	43,433	0.98 (0.96, 0.99)	0.98 (0.97, 0.99)
Red squirrel (*Tamiasciurus hudsonicus*)	21,586	0.85 (0.84, 0.96)	0.86 (0.88, 0.97)
River otter (*Lontra canadensis*)	1,821	0.96 (0.92, 0.98)	0.97 (0.93, 0.98)
Snowshoe hare (*Lepus americanus*)	37,467	0.97 (0.94, 0.99)	0.97 (0.95, 0.98)
Steller's jay (*Cyanocitta stelleri*)	1,844	0.91 (0.8, 0.98)	0.96 (0.87, 1)
Striped skunk (*Mephitis mephitis*)	12,416	0.98 (0.9, 0.99)	0.97 (0.96, 0.98)
Swift fox (*Vulpes velox*)	3,266	0.85 (0.81, 0.88)	0.95 (0.92, 0.97)
Sylvilagus family	6,385	0.93 (0.82, 0.99)	0.94 (0.86, 0.97)
Totals	2,682,380	0.97	0.97
Vehicle (truck, ATV, car)	32,912	0.97 (0.96, 0.98)	0.97 (0.97, 0.98)
White‐tailed deer (*Odocoileus virginianus*)	88,531	0.93 (0.83, 1)	0.97 (0.84, 0.99)
Wild pig (*Sus scrofa*)	243,344	0.98 (0.98, 0.99)	0.99 (0.98, 1)
Wild turkey (*Meleagris gallopavo*)	15,686	0.94 (0.88, 0.99)	0.98 (0.95, 1)
Wolf (*Canis lupus*)	3,070	0.96 (0.88, 1)	0.95 (0.8, 1)
Wolverine (*Gulo gulo*)	18,810	0.98 (0.96, 1)	0.98 (0.97, 0.99)

**TABLE 3:** Out‐of‐sample validation results. All out‐of‐sample images are available from lila.science/datasets

Dataset	Number of images tested	Model tested	Accuracy	Top‐5 accuracy^a^
Snapshot Karoo (South Africa)	38,101	Empty‐animal	0.906	
Snapshot Serengeti (Tanzania)	104,651	Empty‐animal	0.941	
Wellington (New Zealand)	266,966	Empty‐animal	0.939	
Caltech Camera Traps (USA)	218,147	Species	0.562	0.744
ENA24‐Detection (USA)	5,285	Species	0.507	0.649
Missouri Camera Traps (USA)	5,008	Species	0.363	0.652
Saskatchewan (Canada)	5,200	Species	0.913	0.938

^a^ Top‐5 accuracy is not relevant for the empty‐animal model because there are only two classes.

## 4 DISCUSSION

**FIGURE 4:**
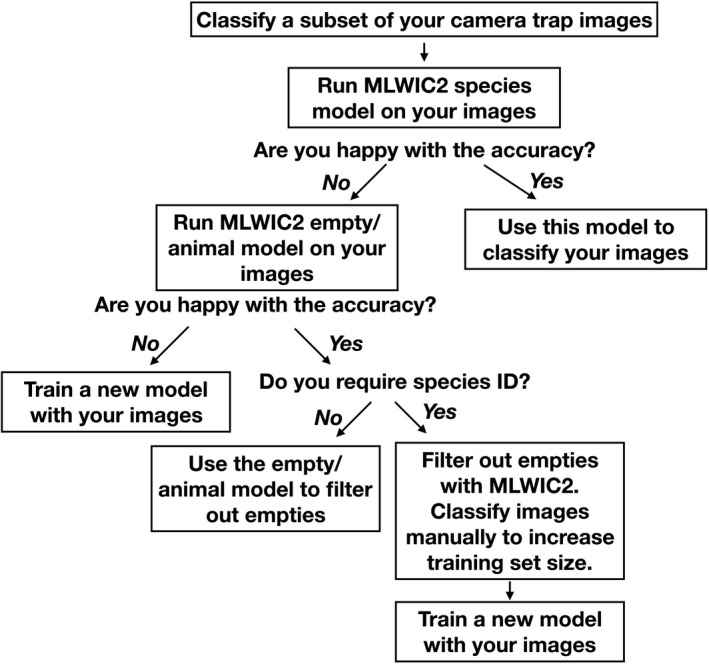
Proposed workflow for using MLWIC2 models when classifying camera trap images

In MLWIC2, we provide two trained machine learning models, one classifying species and another distinguishing between images with animals and those that are empty, with 97% accuracy, which can potentially be used to rapidly classify camera trap images from many locations. While the species model performed well on out‐of‐sample images from Saskatchewan, Canada (91% overall accuracy), the model performed poorly on some out‐of‐sample datasets (Table 3; Figure 2). The discrepancy in model performance on images from different datasets indicates that transferability remains an issue and our species model will not be useful on all datasets; some users will need to train new models on images from their field sites, an option that is available in MLWIC2. Nevertheless, even in the Missouri dataset where our model performed worst, the top‐5 accuracy, the rate at which the true species in an image was in the model's top‐5 guesses, was 65% (Table 3). For some applications, for example, detection of invasive or rare species, such an out‐of‐sample top‐5 recall rate may be sufficient to address scientific questions or meet monitoring objectives. Additionally, our empty‐animal model performed well at distinguishing empty images from those containing animals in datasets from three different countries (91%–94% accuracy), indicating that this model may be broadly applicable for finding empty images in datasets globally. For many research projects, the task of simply removing empty images can save thousands of hours of labor. We propose a workflow for how users can apply these models to filter‐out empty images and train new models as necessary (Figure 4). By providing Shiny Applications to train models and classify images, we make this technology accessible to more scientists with minimal programming experience. Our finding that high recall (>95%) can be achieved with fewer than 2,000 images for some species (Table 2; Figure 1) suggests that smaller labeled image datasets can potentially be used to train models with this software.

Other researchers have developed models for recognizing animals in camera traps, with some success in out‐of‐sample identification. For example, Zilong software accurately removed 85% of empty images (Wei et al., 2020), MegaDetector had a precision of 89%–99% at detecting animals (Beery et al., 2019), and MLWIC achieved an accuracy of 82% at out‐of‐sample species classification (Tabak et al., 2018, 2019). We hypothesize that our models performed well on some out‐of‐sample datasets (Snapshot Serengeti, Snapshot Karoo, Wellington, and Saskatchewan; Table 3) because they were trained using camera trap images from multiple locations with different camera placement protocols, allowing the model to develop a search image for each species in multiple backgrounds (Figure 3).

Transferability of machine learning models remains a complication for implementing these models more broadly to camera trap data and, in many cases, it is most productive for scientists to build models that are trained directly on their study sites (see Figure 4 for more details). While such models will have less broad applicability (they are unlikely to be accurate globally), they can have high study‐specific accuracies, thus reducing the burden of manual image classification. Our finding that models become more generalizable when more datasets are used to train the model (Figure 3) indicates that by including more diverse datasets when we train future models, we may be able to train a model that can be accurate in more locations.

### 4.1 Future directions

As this new technology becomes more widely available, ecologists will need to decide how it will be applied in ecological analyses. For example, when using machine learning model output to design occupancy and abundance models, we can incorporate accuracy estimates that were generated when conducting model testing. The error of a machine learning model in identifying species from camera traps is similar to the problem of imperfect detection of wildlife when conducting field surveys (McIntyre, Majelantle, Slip, & Harcourt, 2020). Wildlife are often not detected when they are present (false negatives) and occasionally detected when they are absent (false positives); ecologists have developed models to effectively estimate occupancy when data have these types of errors (Guillera‐Arroita, Lahoz‐Monfort, van Rooyen, Weeks, & Tingley, 2017; Royle & Link, 2006). We can use Bayesian occupancy and abundance models where the central tendencies of the prior distributions for the false negative and false‐positive error rates are derived from validation of our machine learning models. While we would expect false‐positive rates in occupancy models to resemble the false‐positive error rates for the machine learning model, false‐negative error rates would be a function of the both the machine learning model and the propensity for some species to avoid detection by cameras when they are present (Tobler, Zúñiga Hartley, Carrillo‐Percastegui, & Powell, 2015).

Another area in need of consideration is how to group taxa when few images are available for the species. We generally grouped species when few images were available for model training using an arbitrary cut off of approximately 1,000 images per group (Table 2). Nevertheless, we had relatively few images of grizzly bears (*Ursus arctos horribilis*; *n* = 843), but we included this species because it is of conservation concern, and found high rates of recall and precision (99% for each). We grouped members of Mustelidae (*Mustela erminea*, *Mustela frenata*, unknown *Mustela* spp., *Neovison* spp., and *Taxidea taxus*) together, and this group had relatively low recall and precision (89% and 91%, respectively). When researchers develop new models and decide which species to include and which to group, they will need to consider the available data, the species or groups in their study, and the ecological question that the model will help address.

## CONFLICT OF INTEREST

The authors have no conflicts of interest to declare.

## AUTHOR CONTRIBUTION

**Michael A Tabak:** Conceptualization (lead); Data curation (equal); Formal analysis (lead); Investigation (lead); Methodology (lead); Project administration (equal); Software (lead); Validation (lead); Visualization (equal); Writing‐original draft (lead); Writing‐review & editing (lead). **Mohammad Sadegh Norouzzadeh:** Formal analysis (equal); Methodology (equal); Software (equal); Writing‐review & editing (equal). **David Wolfson:** Data curation (lead); Writing‐review & editing (equal). **Erica Newton:** Software (equal); Writing‐review & editing (equal). **Raoul Boughton:** Data curation (equal); Funding acquisition (equal); Writing‐review & editing (equal). **Jacob Ivan:** Data curation (equal); Writing‐review & editing (equal). **Eric A Odell:** Data curation (equal); Writing‐review & editing (equal). **Eric S Newkirk:** Data curation (equal); Writing‐review & editing (equal). **Reesa Conrey:** Data curation (equal); Writing‐review & editing (equal). **Jennifer Leigh Stenglein:** Data curation (equal); Writing‐review & editing (equal). **Fabiola Iannarilli:** Data curation (equal); Writing‐review & editing (equal). **John D. Erb:** Data curation (equal); Writing‐review & editing (equal). **Ryan Kendall Brook:** Data curation (equal); Writing‐review & editing (equal). **Amy Davis:** Data curation (equal); Writing‐review & editing (equal). **Jesse S Lewis:** Data curation (equal); Writing‐review & editing (equal). **Daniel Walsh:** Data curation (equal); Writing‐review & editing (equal). **James Beasley:** Data curation (equal); Writing‐review & editing (equal). **Kurt Vercauteren:** Conceptualization (equal); Data curation (equal); Writing‐review & editing (equal). **Jeff Clune:** Methodology (supporting); Software (supporting); Writing‐review & editing (equal). **Ryan S Miller:** Conceptualization (equal); Funding acquisition (lead); Project administration (equal); Visualization (lead); Writing‐original draft (supporting); Writing‐review & editing (equal).

## AUTHOR CONTRIBUTIONS

MAT, RSM, and RKBoughton conceived of the project. DWW, RKB, JSI, EAO, ESN, RYC, JLS, FI, JE, RKB, AJD, JSS, DPW, JCB, and KCV oversaw the data collection and labeling processes. MSN and JC provided insight for model training. MAT developed MLWIC2 and led the writing of the manuscript. DWW and EJN assisted with MLWIC2 development. All authors contributed critically to drafts and gave final approval for submission.

## Supporting information

Appendix S1

Appendix S2

Appendix S3

Appendix S4

## Data Availability

The trained models described in this work are available in the MLWIC2 package (https://github.com/mikeyEcology/MLWIC2). Images used to train models are available in the North American Camera Trap Images dataset (lila.science/datasets/nacti). Data from validation tests are available from the dryad digital repository (https://doi.org/10.5061/dryad.x95x69pfx; Tabak, 2020).
