# Supplementary material for: Improving the accessibility and transferability of machine learning algorithms for identification of animals in camera trap images: MLWIC2
Source: Ecol Evol. 2020 Sep 16;10(19):10374–83. doi: 10.1002/ece3.6692 (PMC7548173; doi:10.1002/ece3.6692)
Supplement: Supplementary file 3 — Appendix S3 [file ECE3-10-10374-s003.docx]

Appendix S3: Calculation of pooled recall and precision rate and corresponding confidence intervals

Results of MLWIC2 for each study were pooled to estimate an overall recall and precision rate and corresponding confidence intervals. To achieve this the R package ‘General Package for Meta-Analysis’ (meta v4.9-6) was used (Schwarzer, 2020; Schwarzer et al 2015).

A random intercept logistic regression model was utilized to pool results from each study due to the very large differences in sample sizes (Stijnen et al., 2010, Schwarzer et al., 2019). Based on suggestions from Warton & Hui (2011) the logit transformation was used in fitting the models instead of the arcsine transformation. Additionally, the logit transformation allowed individual study weights to be considered when estimating the pooled estimates for recall and precision. A continuity correction of 0.5 was applied to studies with zero cell frequencies - that is when a species was always identified correctly.

**References**

Schwarzer, G., Chemaitelly, H., Abu‐Raddad, L.J. and Rücker, G., 2019. Seriously misleading results using inverse of Freeman‐Tukey double arcsine transformation in meta‐analysis of single proportions. Research synthesis methods, 10(3), pp.476-483.

Stijnen, T., Hamza, T.H. and Özdemir, P., 2010. Random effects meta‐analysis of event outcome in the framework of the generalized linear mixed model with applications in sparse data. Statistics in medicine, 29(29), pp.3046-3067.

Schwarzer, G. 2020. Meta: general package for meta-analysis (version 4.9-6).

Schwarzer, G., Carpenter, J.R. and Rücker, G., 2015. Meta-analysis with R (Vol. 4784). Cham: Springer.

Warton, D.I. and Hui, F.K., 2011. The arcsine is asinine: the analysis of proportions in ecology. Ecology, 92(1), pp.3-10.

Disclaimer: Any use of trade, firm, or product names is for descriptive purposes only and does not imply endorsement by the U.S. Government.
